# An Atypical Autoinflammatory Disease Due to an LRR Domain NLRP3 Mutation Enhancing Binding to NEK7

**DOI:** 10.1007/s10875-021-01161-w

**Published:** 2021-10-21

**Authors:** Emily A. Caseley, Samuel Lara-Reyna, James A. Poulter, Joanne Topping, Clive Carter, Fatima Nadat, Gavin P. Spickett, Sinisa Savic, Michael F. McDermott

**Affiliations:** 1 Leeds Institute of Rheumatic and Musculoskeletal Medicine, St James’s University Hospital, Leeds, UK; 2 Institute of Microbiology and Infection, University of Birmingham, Birmingham, UK; 3 Transplant and Cellular Immunology, St James’s University Hospital, Leeds, UK; 4 Regional Department of Immunology, Royal Victoria Infirmary, Newcastle Upon Tyne, UK; 5 Department of Clinical Immunology and Allergy, St James’s University Hospital, Leeds, UK; 6 National Institute for Health Research–Leeds Biomedical Research Centre, Chapel Allerton Hospital, Leeds, UK

**Keywords:** NLRP3 inflammasome, *NLRP3*-associated autoinflammatory diseases, Leucine-rich repeat domain, Inflammation, NIMA-related kinase 7

## Abstract

The NLRP3 inflammasome is a vital mediator of innate immune responses. There are numerous *NLRP3* mutations that cause *NLRP3*-associated autoinflammatory diseases (*NLRP3*-AIDs), mostly in or around the NACHT domain. Here, we present a patient with a rare leucine-rich repeat (LRR) domain mutation, p.Arg920Gln (p.R920Q), associated with an atypical *NLRP3*-AID with recurrent episodes of sore throat and extensive oropharyngeal ulceration. Unlike previously reported patients, who responded well to anakinra, her oral ulcers did not significantly improve until the PDE4 inhibitor, apremilast, was added to her treatment regimen. Here, we show that this mutation enhances interactions between NLRP3 and its endogenous inhibitor, NIMA-related kinase 7 (NEK7), by affecting charge complementarity between the two proteins. We also demonstrate that additional inflammatory mediators, including the NF-кB and IL-17 signalling pathways and IL-8 chemokine, are upregulated in the patient’s macrophages and may be directly involved in disease pathogenesis. These results highlight the role of the NLRP3 LRR domain in *NLRP3*-AIDs and demonstrate that the p.R920Q mutation can cause diverse phenotypes between families.

**Supplementary Information:**

The online version contains supplementary material available at 10.1007/s10875-021-01161-w.

## Introduction

NLRP3 is an integral part of the NLRP3 inflammasome, a large cytosolic protein complex which acts as an intracellular innate immune sensor. NLRP3 inflammasome assembly occurs following the detection of a wide range of harmful stimuli and mediates caspase-1 autoproteolysis and activation, leading to caspase-1-mediated cleavage of the pro-inflammatory cytokines, IL-1β and IL-18, into their active forms, and cleavage of gasdermin-D, which subsequently forms membrane pores and induces pyroptotic cell death. Gain-of-function mutations in *NLRP3* are associated with a series of rare autoinflammatory diseases (AIDs), collectively known as *NLRP3*-associated autoinflammatory diseases (*NLRP3*-AIDs), previously referred to as cryopyrin-associated periodic syndromes (CAPS) [1]. *NLRP3*-AIDs are typified by recurrent episodes of fever, urticaria, arthralgia, and chronic inflammation which can lead to long-term damage including sensorineural hearing loss and amyloidosis [2].

The NLRP3 protein comprises three domains: the pyrin domain (PYD), the central NACHT domain, and the leucine-rich repeat (LRR) domain. Most of the reported *NLRP3*-AID-related *NLRP3* mutations are located in or around the central NACHT domain [3], whereas the C terminus LRR domain, the function of which is unclear due to evidence that it is dispensable for NLRP3 inflammasome activation [4], is an uncommon site for pathogenic mutations [3]. One of the few validated LRR domain mutations is the missense substitution c.2759G > A, coding for p.Arg920Gln (p.R920Q) in the LRR domain. This mutation was previously reported in two unrelated North American families, where it was previously known as p.Arg918Gln (p.R918Q) and primarily caused autosomal-dominant sensorineural hearing loss [5] which is common in *NLRP3*-AIDs [6]. However, the symptoms observed in these families were varied; one family reported hearing loss with an age of onset in the second to fourth decade of life, with no additional physical signs or symptoms of *NLRP3*-AID. In contrast, the second family experienced hearing loss in the first 10 years which was accompanied by autoinflammatory signs and symptoms, including oral ulcers, without serologic signs of inflammation, contributing to an atypical *NLRP3*-AID phenotype [5]. Hearing loss and autoinflammatory symptoms were generally reversed or improved following treatment with the interleukin-1 (IL-1) receptor antagonist anakinra, which has been historically used in the treatment of *NLRP3*-AIDs [7–9]. Here, we present another case of a patient with the *NLRP3*-p.R920Q mutation. This patient’s symptoms spanned the spectrum of those seen in this previous study; she experienced mild hearing loss (only evident on audiogram) with some evidence of systemic inflammation, but her main complaint was persistent oropharyngeal ulcers. She showed only a partial response to anakinra, instead responding well to steroids and the phosphodiesterase type-4 (PDE4) inhibitor apremilast.

In this report, we investigated this atypical patient phenotype, the potential pathogenic mechanisms of this LRR domain mutation and signalling pathways which contribute to the disease. Altogether, our study provides a detailed exploration of this rare pathogenic mutation.

## Methods

### Human Subjects

Work using human samples was approved by the Health Research Authority (study number 18/YH/0070). Informed written consent was obtained from participants prior to sample collection. Age- and sex-matched HCs were recruited from St James’s University Hospital, Leeds, UK.

### Samples

Patient and HC samples were collected in VACUETTE® tubes (Greiner Bio-One), coated with EDTA anticoagulant or serum clot activator gel for whole blood or serum, respectively. Serum samples were collected by allowing the sample to clot for 1 h, followed by centrifugation at 1000 × *g* for 15 min.

### Sequencing

The coding sequence was captured from genomic DNA using the SureSelectXT target enrichment kit with All Exon v5 capture library (Agilent). Sequencing was performed on a HiSeq 3000 (Illumina) using a 2 × 150-bp paired-end sequencing protocol, and analysis restricted to the autoinflammatory/autoimmune gene panel (supplementary methods).

### Cell Culture and Stimulation

HEK293T cells (ATCC) were cultured according to the manufacturer’s specifications. Monocytes were isolated from whole blood using Lymphoprep density gradient media (StemCell), as outlined in the supplementary methods. The NLRP3 inflammasome was stimulated with 10 ng/mL LPS (Ultrapure Escherichia coli K12, Invivogen) for 4 h, with 5 mM ATP (Invivogen) added for the last 30 min. After stimulation, cells were lysed with TRIzol (Invitrogen).

### Cytokine Quantification by ELISA

Release of IL-1β, IL-18, TNF, IL-6, or IL-10 in patient sera or media from cultured cells was detected by enzyme-linked immunosorbent assay (ELISA). Nunc MaxiSorp 96-well plates (Thermo Fisher Scientific) and commercially available ELISA kits were used (Thermo Fisher Scientific, product codes listed in supplementary methods) according to the manufacturer’s specifications.

### ASC Speck Quantification by Flow Cytometry

A 2 μL of PE-conjugated ASC antibody (HASC-71 clone, BioLegend) was added to 100 μL of cell culture media, and the mixture incubated in FACS collection tubes on a shaker for 1 h. Size gating was carried out with Megamix-Plus beads (Biocytex) according to the manufacturer’s specifications and was used to threshold out readings below 0.9 μm (representative flow cytometry plots shown in supplementary methods). Samples were run and analysed on a CytoFLEX-S (Beckman Coulter).

### Gene Expression Analysis

RNA isolation was carried out using TRIzol Reagent and Phasemaker tubes (Thermo Fisher Scientific) according to the manufacturer’s specifications. One hundred nanograms of RNA was converted to cDNA using the SuperScript IV one-step RT-PCR system (Invitrogen) according to the manufacturer’s specifications. Gene expression was analysed using TaqMan probes (Thermo Fisher Scientific); the supplementary methods detail the probes and cycle parameters used. Data were expressed as relative expression compared to the housekeeping genes, *ACTB* and *HPRT1.*

### RNA Preparation and RNAseq

RNA was isolated using TRIzol (Invitrogen) and Phasemaker tubes (Thermo Fisher Scientific) according to the manufacturer’s instructions. RNA quality was assessed using an Agilent 4200 TapeStation (Agilent Technologies). Library preparation and RNA-seq was carried out on a high-throughput Illumina platform and paired-end reads generated (Novogene (UK) Company Limited); further information is provided in the supplementary methods.

### *In Silico* Investigation

The cryo-EM structure of NLRP3 in complex with NEK7 (PDB 6NPY)[10] was used for structural investigations. Interaction analysis was conducted using PISA [11] and structure representations, including electrostatic surface representations, produced using Pymol version 2.3.3 [12].

### Immunoprecipitation Studies

Cells were transfected with 10 μg of plasmids for NLRP3-GFP and/or NEK7-His (details in supplementary methods) using Opti-MEM and Lipofectamine 2000 (Thermo Fisher Scientific) according to the manufacturer’s specifications. Cell lysates were harvested and used for immunoprecipitation, then analysed by SDS-PAGE and Western blot.

### Statistical Analysis

Data were presented as the mean ± standard error of the mean (SEM). Analyses were performed using GraphPad Prism 9. A two-way ANOVA test with Tukey’s post-hoc analysis was performed when calculating variance between samples (*p* values *p < 0.05, **p < 0.01, ***p < 0.001). *p* < 0.05 was considered significant.

## Results

### Case Presentation

This female patient presented at the age of 9 years mainly with recurrent episodes of sore throat and associated extensive oropharyngeal ulceration. She also reported episodic fevers but without any clear pattern. Other reported problems included recurrent chest infections treated with antibiotics, painful joints (particularly knees and hands), headaches associated with sickness and vomiting, and episodes of periorbital cellulitis. There was no history of genital ulceration, other skin rashes, unexplained abdominal or chest pains or previous thromboembolic events. She did not report any hearing difficulties. Prior to the genetic diagnosis, there was no overt family history of similar problems. She was extensively investigated, and her immunological workup showed a normal immunoglobulin and lymphocyte profile. Tests of her lymphocyte and neutrophil function also showed no obvious abnormalities. The only positive finding was a non-specific speckled anti-nuclear antibody test, but she had no other clinical features for a diagnosis of connective tissue disease. She did not meet the diagnostic criteria for Behcet’s disease, and she was HLA-B51 negative. Interestingly, the systemic inflammatory marker, C-reactive protein, was not always elevated during acute flairs. Over the 15 years following her initial presentation, she continued to be symptomatic despite numerous trials of different treatments. She responded well to corticosteroids, which controlled her oropharyngeal ulceration, but did not benefit from colchicine, hydroxychloroquine, azathioprine, methotrexate, infliximab, tocilizumab or baricitinib. She had a trial of anakinra (up to 200 mg daily), which helped with fevers, and to some extent headaches and arthralgia, but had no effect on the mucosal ulceration. Finally, apremilast was added to her anakinra regimen, and this combination led to significantly better symptomatic control with gradual tapering and eventual discontinuation of corticosteroids. She subsequently became pregnant which necessitated withdrawal of apremilast since this medication is not licenced in pregnancy. Consequently, she relapsed with oropharyngeal ulceration becoming troublesome and requiring reintroduction of high-dose prednisolone.

### The Inflammatory Phenotype Is Associated with an LRR Domain NLRP3 R920Q Mutation

**Fig. 1:**
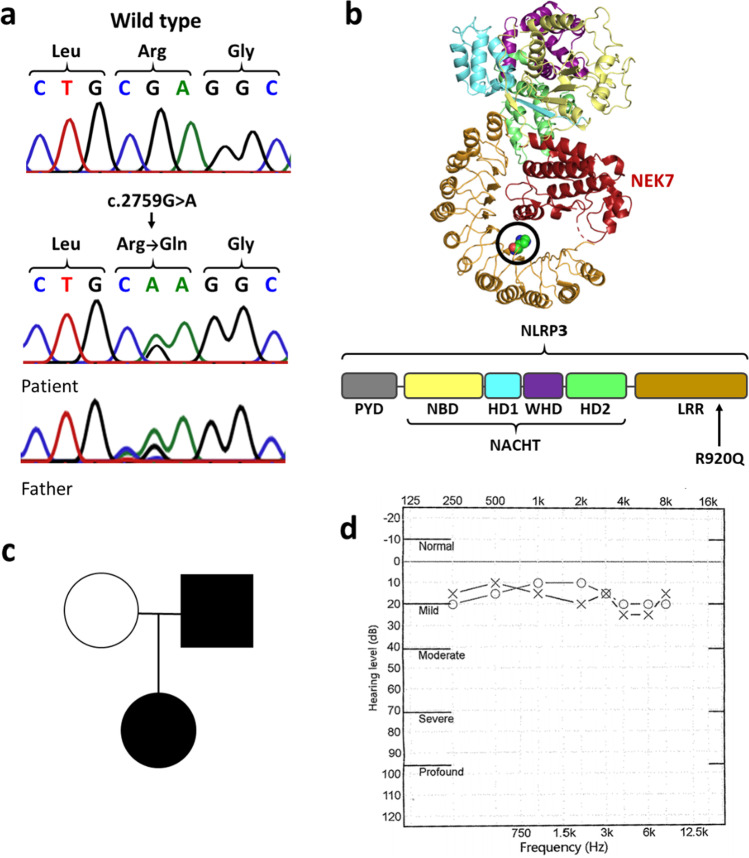
The autosomal dominant p.R920Q *NLRP3* mutation is associated with an enhanced inflammatory phenotype. **a** Sequence chromatogram showing the wild-type NLRP3 sequence (top) and the c.2759G > A transition, leading to the p.R920Q mutation in the patient and patient’s father as indicated (bottom). **b** A 3D representation of the NLRP3 structure in complex with its regulator NEK7, with the pyrin domain and N-lobe omitted from NLRP3 and NEK7, respectively. Domains are colour coded as shown in the 2D representation, with domains omitted from the structures shown in grey. The R920 residue is circled in black. **c** p.R920Q inheritance and family phenotype, hearing loss and periodic fevers. **d** The patient’s audiogram showing mild hearing loss

The patient consented to genetic testing, and exome sequencing of the patient’s genomic DNA was undertaken; variants in a panel of 49 autoinflammatory/autoimmune disease genes were analysed (see Methods). A variant in *NLRP3* was identified, c.2759G > A which is predicted to cause the missense substitution p.Arg920Gln (p.R920Q) (Fig. 1a), which is located in the LRR domain of NLRP3 (Fig. 1b). The variant has a CADD score of 21.5 (v1.6), is not present in approximately 125,000 exomes available in the Genome Aggregation Database (accessed 07.01.21)[13], and has only previously been reported in two unrelated North American families who presented with hearing loss and mixed symptoms of systemic autoinflammation [5]. An autosomal dominant inheritance pattern was also observed in these prior cases, and targeted sequencing of our patient’s parents indicated that the p.R920Q mutation was inherited from her father, who also had a history of hearing loss (Fig. 1a, c). No systemic inflammation was reported by the patient’s father, although limited information about him was available. The patient experienced hearing loss, although this was mild to the extent that she did not report it and it only became apparent in the audiogram (Fig. 1d).

### Heightened Inflammatory Responses in Primary Cells from the Patient

**Fig. 2:**
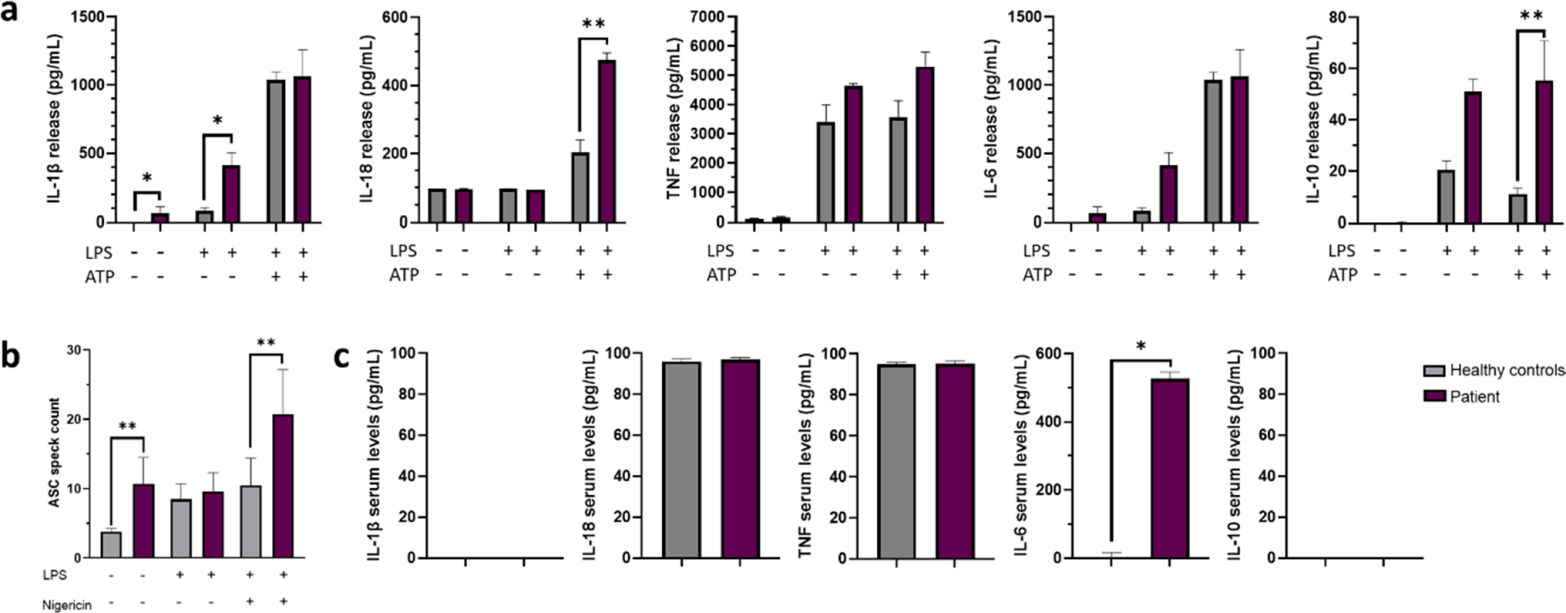
The NLRP3 p.R920Q mutation leads to a *NLRP3*-AID cytokine release profile**. a** Cytokine profile of patient’s primary monocytes when unstimulated, stimulated with LPS alone or with LPS and ATP for NLRP3 inflammasome activation, compared to 3 healthy controls (HCs). **b** Extracellular ASC speck levels detected by flow cytometry in cell media supernatants from patients’ or HC monocytes which were unstimulated, stimulated with LPS alone or stimulated with both LPS and nigericin for NLRP3 inflammasome activation **c**. Cytokine levels in patient and HC serum. Data shown is compiled from 3 independent experiments and is presented as mean ± SEM, *n* = 3. *p < 0.05, **p < 0.01, ***p < 0.001

The NLRP3 inflammasome is primarily expressed by myeloid cells [14]; thus, we assessed the patient’s monocyte responses to canonical NLRP3 inflammasome stimulation in vitro using lipopolysaccharide (LPS) and extracellular ATP. Basal IL-1β levels were significantly elevated compared to healthy control (HCs), and stimulation by LPS alone caused significantly higher IL-1β release from patient monocytes compared to HCs, which is indicative of an *NLRP3*-AID [15] (Fig. 2a). In contrast, there was no notable difference in maximal IL-1β release from patient and HC monocytes in response to LPS and ATP stimulation, although patient monocytes stimulated for the NLRP3 inflammasome released significantly higher levels of IL-18 compared to HCs. *Il1b* gene expression was also higher in patient’s monocytes compared to HCs when unstimulated or stimulated with LPS only, but not with LPS and ATP (Fig. S1a). Like IL-1β, IL-6 release from the patient’s monocytes in response to LPS stimulation alone was elevated compared to HCs, albeit not significantly, whereas release of another proinflammatory cytokine which is not directly regulated by the NLRP3 inflammasome, TNF, was similar between patients’ and HC monocytes under all conditions. In contrast, the anti-inflammatory cytokine IL-10 was significantly elevated following stimulation with LPS alone (Fig. 2a). In contrast, TNF release from the patient’s M1 macrophages was significantly higher than from HCs (Fig. S1b). Amplified NLRP3-mediated cytokine release has been associated with an increase in the formation of ASC specks, micrometre-sized structures formed by the adaptor protein ASC (apoptosis-associated speck-like protein containing a CARD) [16–19]. Significantly higher levels of extracellular ASC specks were detected in cell culture media from unstimulated or LPS and ATP-stimulated patient’s monocytes compared to HCs, suggesting that this mutation allows the NLRP3 inflammasome complex to form more readily (Fig. 2b). In contrast to cell supernatants, determination of circulating cytokine levels in the patient’s serum showed no significant difference in IL-1β, IL-18, TNF or IL-10 levels compared to HC serum. However, IL-6 was significantly raised in the patient’s serum (Fig. 2c), as previously observed in different *NLRP3*-AIDs [8, 20, 21].

### The NLRP3 p.R920Q Mutation Enhances NLRP3-NEK7 Interactions

Our initial assays support previous reports of p.R920Q being a pathogenic *NLRP3* mutation [22, 23]. To explore the mechanism(s) whereby this mutation affects NLRP3 inflammasome function, we interrogated the recently determined cryo-electron microscopy (cryo-EM) structure of NLRP3 in complex with NEK7 [10]. Residue 920 lies in the LRR domain at the NLRP3/NEK7 interface (Fig. 1b), and previously reported LRR domain mutations affect specific interactions between NLRP3 and NEK7 [24–26]; we therefore investigated the effects of the p.R920Q mutation on NLRP3/NEK7 interactions.

**Fig. 3:**
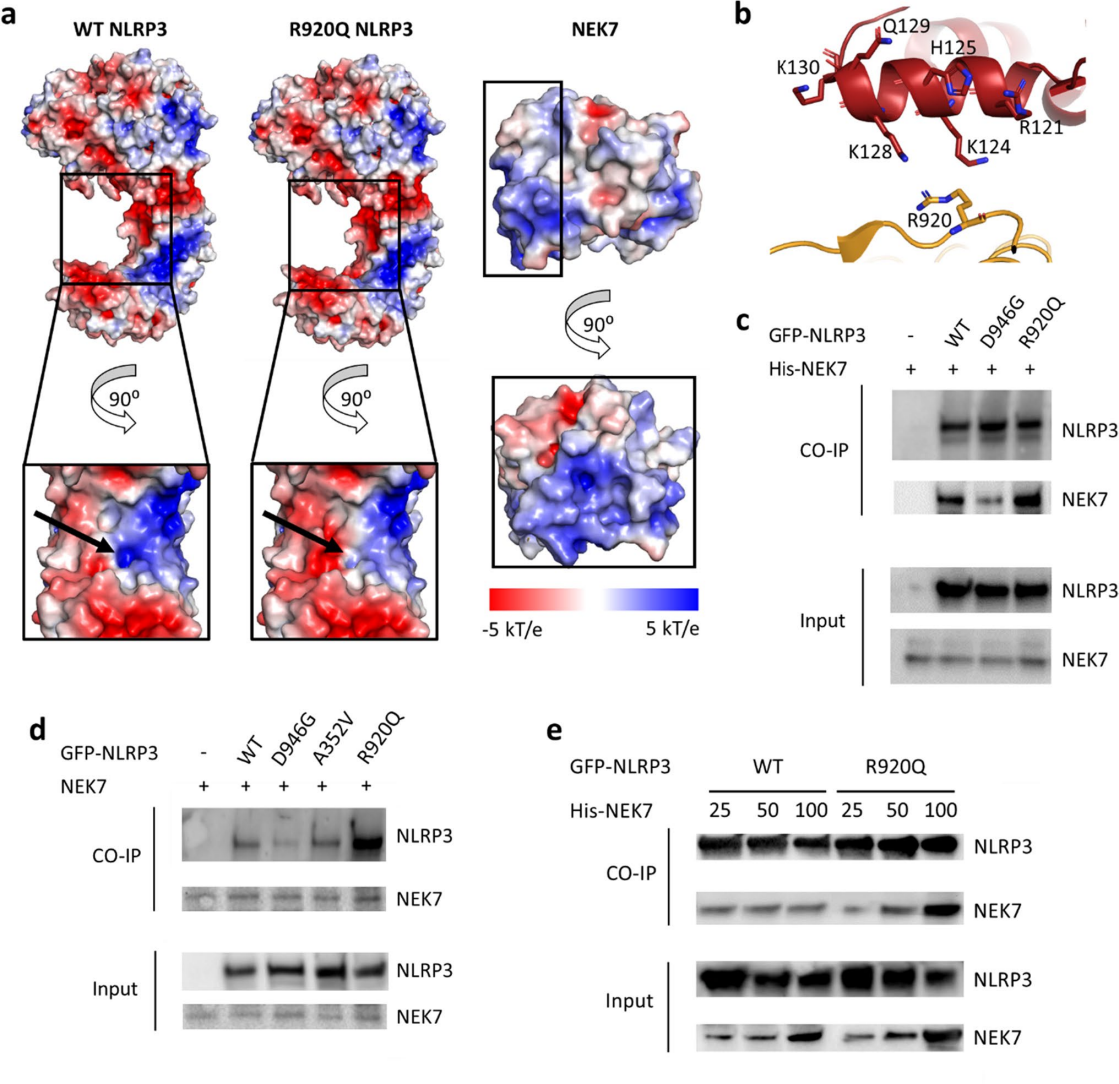
The p.R920Q mutation in the NLRP3 protein enhances NLRP3-NEK7 interactions. **a** Electrostatic surface representation of WT NLRP3 (left), NLRP3 with the p.R920Q mutation (centre) and NEK7 (right); electrostatic potential is coloured to show negative charge (− 5.0 kT e^−1^) in red and positive charge (5.0 kT e^−1^) in blue, and black arrows show the change in charge density resulting from the p.R920Q mutation. Black boxes outline interacting domains. **b** Magnified view of the interface between NLRP3 (orange) and NEK7 (red) surrounding residue 920. The main chains are shown as cartoons and potentially interacting side chains as sticks. **c** NLRP3-NEK7 association analysed by immunoprecipitation and immunoblot in lysates of HEK293T cells transfected with His-tagged NEK7 alone, or co-transfected with WT NLRP3 or NLRP3 containing the p.D926G or p.R920Q mutations. Representative blots are shown of three separate experiments; replicates are shown in Fig. S2. **d** NLRP3-NEK7 association analysed by immunoprecipitation and immunoblot in lysates of HEK293T cells expressing endogenous NEK7 and transfected with either WT or mutant NLRP3 as indicated; replicates are shown in Fig. S3. **e** Immunoprecipitation and immunoblot assays in lysates of HEK293T cells co-transfected with WT or p.R920Q-NLRP3 with increasing concentrations of His-tagged NEK7; 25%, 50% or 100% as indicated; replicates are shown in Fig. S4. Immunoprecipitation and immunoblot assays were repeated 3 times in each instance

Interactions between NLRP3 and NEK7 are determined in part by charge complementarity; the calculated isoelectric points (pIs) of NEK7 and NLRP3 are 8.5 and 6.2, respectively, indicating an overall positive charge for NEK7 and overall negative charge for NLRP3 at a physiological pH [10]. However, there is a cluster of positive charge density in the NLRP3 LRR domain at the interface with NEK7 in the region of R920 (Fig. 3a). In silico mutation of this residue to glutamine was predicted to reduce this net positive charge, thereby increasing the electrostatic interactions between the LRR domain and the positively charged NLRP3-interacting surface of NEK7 (Fig. 3a).

Important residues in NEK7 involved in interactions with the NLRP3 LRR domain in the immediate vicinity of R920, as assessed by PISA [11], included R121, K124, H125, K128 and Q129 (Fig. 3b). The p.R920Q mutation was predicted to impact the local confirmation of residues, increasing the buried surface area of K124 by 10% and decreasing that of K128 by 10%, respectively, in addition to increasing the predicted interface stability of the complex [11]. These in silico observations were further interrogated with co-immunoprecipitation experiments using HEK293T cells transfected with WT NLRP3, or NLRP3 with the p.R920Q or the previously described p.D946G mutation, which reduces NLRP3/NEK7 interactions [26]. Introduction of the p.D946G mutation reduced the amount of NEK7, pulled down by NLRP3 compared to the WT protein, whereas NLRP3 harbouring the p.R920Q substitution showed increased binding to NEK7 compared to WT-NLRP3 (Fig. 3c and Fig. S2). Additionally, NLRP3 transfected into HEK293T cells was pulled down by the endogenously expressed NEK7 [26, 27]. These co-immunoprecipitation experiments further demonstrated that NLRP3 with a p.D946G mutation reduced the amount of NLRP3 pulled down by NEK7, whereas p.R920Q increased the amount of protein pulled down; in contrast, *NLRP3*-AID-associated mutation p.A352V, located in the NACHT domain, did not affect the interaction between NLRP3 and NEK7 (Fig. 3d), indicating an LRR-domain specific effect. Furthermore, when varying amounts of NEK7 were transfected into HEK293T cells, the quantity of NEK7 pulled down by NLRP3 varied between the WT and p.R920Q protein. Although the amount of NEK7 pulled down at the lowest NEK7 concentration was comparable for both, the NLRP3-p.R920Q protein showed higher affinity for NEK7 at subsequent, higher amounts of transfected NEK7 (Fig. 3e), demonstrating a potential difference in protein–protein interaction kinetics depending on the ratio of these two proteins. Overall, this indicates that NLRP3/NEK7 interactions in the NLRP3 inflammasome containing the R920Q mutation are increased, which is likely to be facilitated by increased charge complementarity. These enhanced protein–protein interactions suggest that the increased IL-1β and IL-18 release in patients’ monocytes may be due to a low threshold for NLRP3 inflammasome activation.

### Transcriptomic Profile and Enriched Inflammatory Pathways in NLRP3-R920Q Macrophages

As shown above, the patient’s monocytes exhibit dysregulated inflammatory cytokine release indicative of an *NLRP3*-AID. However, unlike previous reported cases [22], our patient responded poorly to anakinra, suggesting that other cellular processes may contribute to symptoms such as oropharyngeal ulcers. We therefore carried out RNA-sequencing (RNA-seq) to investigate the transcriptomic profile of M0, M1 and M2 macrophages derived from CD14 + monocytes, taken from the patient and two HCs, to investigate whether dysregulated macrophage polarisation was a contributing factor, as observed in other autoinflammatory diseases [28–30].

**Fig. 4:**
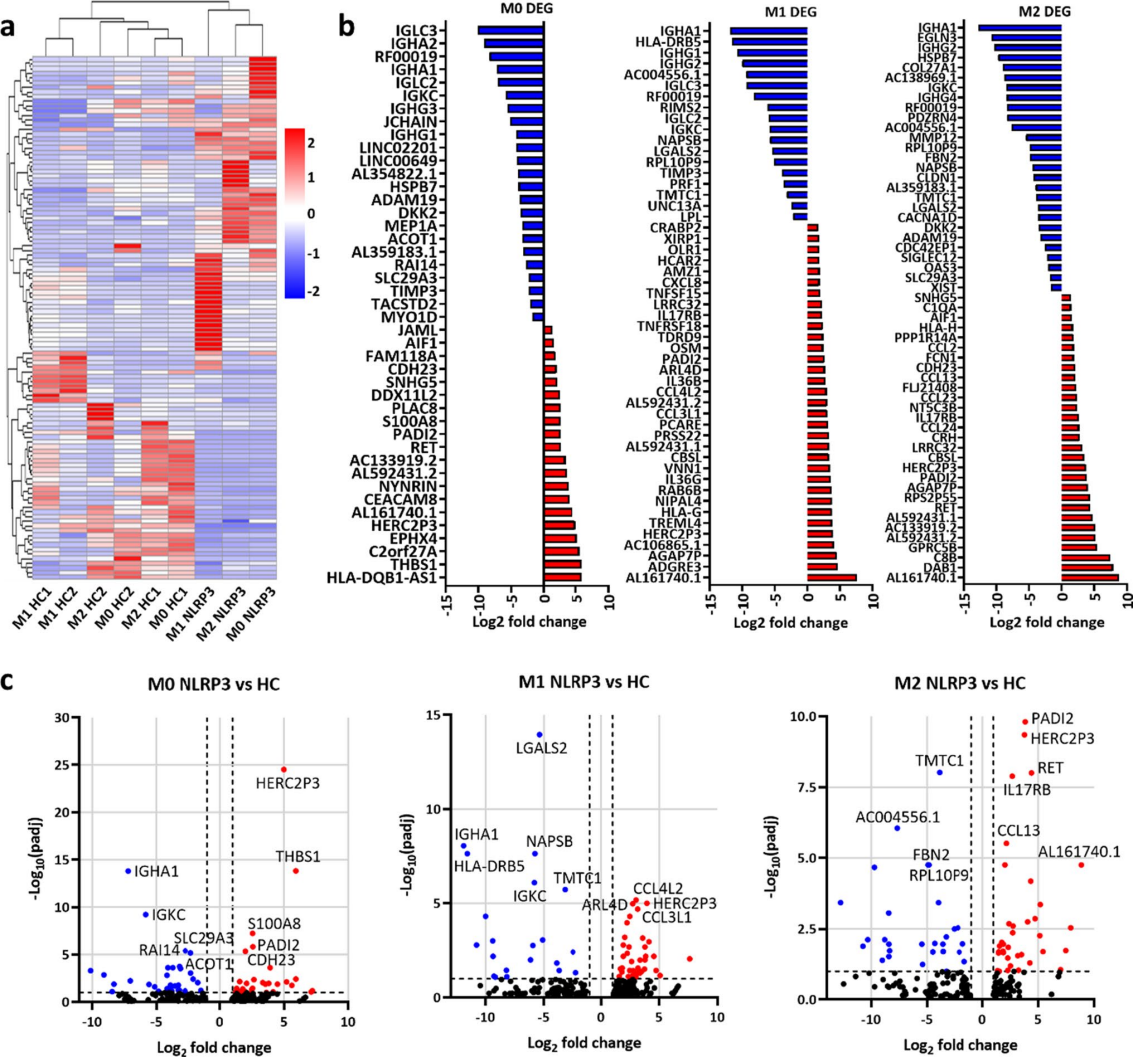
Macrophage transcriptomic profiles differ between patient’s and HCs. **a** Pairwise differential expression between HC and patient’s samples for M0, M1 and M2 macrophages. Heatmaps show the DEGs (*q* < 0.05), and the colour scale represents the Log2(FPKM) values. Genes were grouped by Euclidean clustering in rows. **b** DEGs in M0, M1 and M2 macrophages are shown as Log2-fold change; upregulated genes are shown in red and downregulated genes in blue. **c** Volcano plots showing the Log2-fold change and *p* value for the DEGs in each cell type; DEGs (*p* > 0.05) are shown in blue, if significantly downregulated, and red if significantly upregulated

We initially conducted pairwise comparisons of the differentially expressed genes (DEGs) between the patient’s M0, M1 and M2 macrophages and those from two HCs to investigate the global transcriptomic differences between the different cell types. Even considering the known transcriptomic heterogeneity between donors [31], there was a marked difference in DEGs between cells taken from the patient and from the HCs. Grouping samples by genetic similarity revealed common clusters of DEGs between different macrophage subsets from both HCs, which were largely downregulated in the patient’s macrophages (Fig. 4a). Shared upregulated DEGs between the three cell subsets include the pseudogene *HERC2P3*, encoding the long non-coding RNA HERC2P3 which is related to ubiquitin-protein transferase activity [32], and *PADI2*, encoding peptidyl arginine deiminase 2, which catalyses the post-translational deimination of proteins [33] (Fig. 4b). *HERC2P3* was in the top 10 most significantly up or downregulated DEGs for all 3 macrophage subsets (Fig. 4c). Further notable upregulated DEGs related to inflammatory responses include *IL36B* and *IL36G* in M1 macrophages, which encode the inflammatory cytokines IL-36β and IL-36γ, respectively, and *IL17RB* in M2 macrophages, which encodes IL-17 receptor B which is involved in IL-8 release (Fig. 4b).

**Fig. 5:**
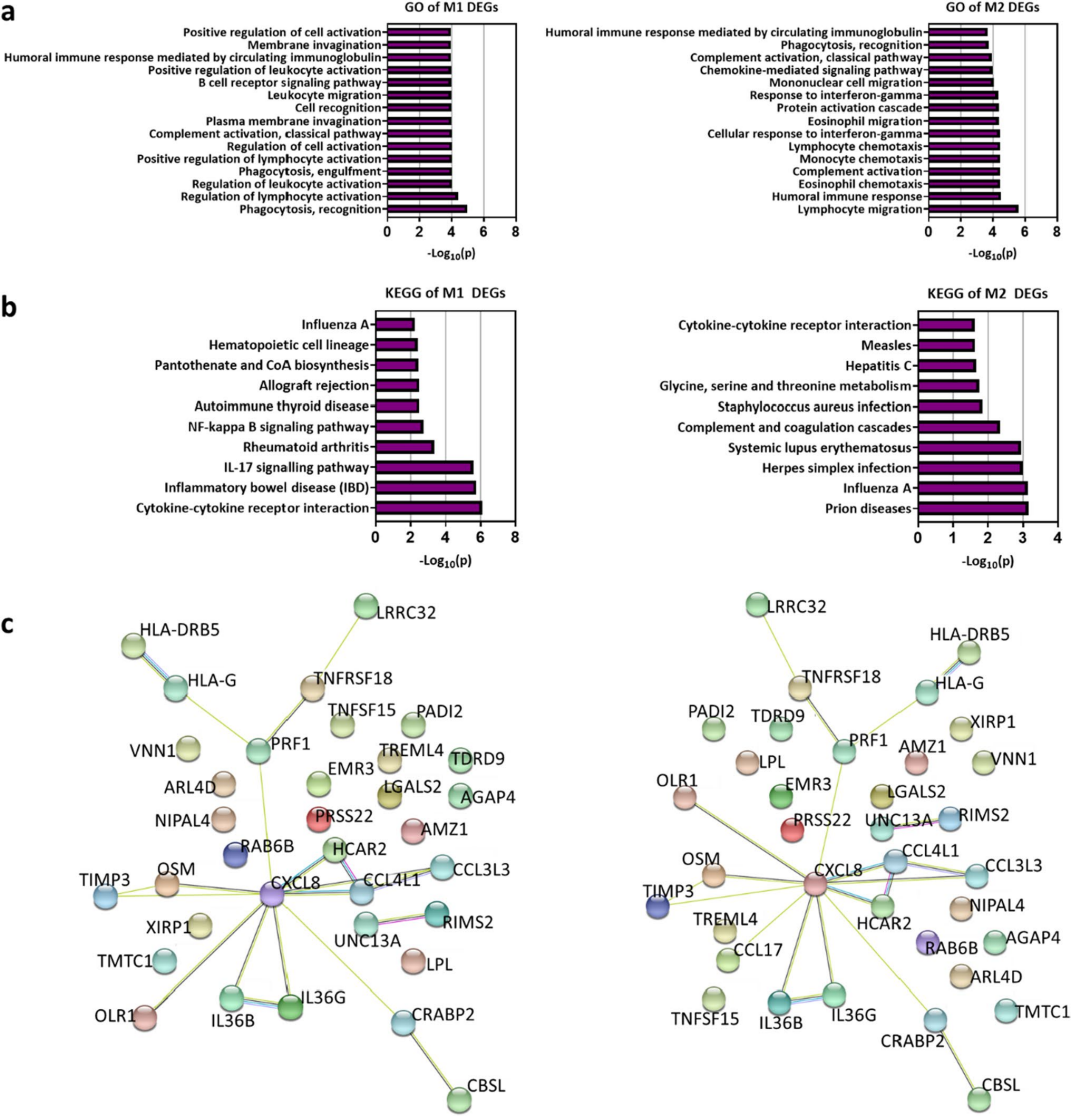
Gene set enrichment analyses reinforce a role for cytokine signalling pathways in the disease state. **a** Gene ontology (GO) enrichment analysis of DEGs between the patient’s and HC macrophages for M1 and M2 macrophages containing 3 sub-ontologies: biological process (BP), cellular component (CC) and molecular function (MF). The 15 most significant GO terms are shown (*p* < 0.001). Enrichment analysis was carried out using DAVID bioinformatics online resource 6.8. **b** Kyoto encyclopaedia of genes and genomes (KEGG) analysis showing biological function and pathway enrichment of DEGs between the patient and HCs for M1 and M2 macrophages (*p* < 0.01). **c** Network of predicted protein–protein interactions derived from the list of DEGs between the patient’s and HC macrophages, as determined by STRING analysis. Lines between nodes depict known interactions from curated databases (light blue), experimentally determined interactions (pink), co-expressed proteins (black) and homologous proteins (violet)

We next analysed the DEGs in our patient’s macrophages compared to the HCs to identify common themes. Gene ontology (GO) analysis [34] showed significant enrichment of several GO terms associated with immune responses (Fig. 5a), although less notably in M0 macrophages (Fig. S5a). However, enriched terms in M2 macrophages included the BP terms ‘response to interferon-gamma’ (GO:0,034,341), ‘cellular response to interferon-gamma’ (GO:0,071,346) and ‘complement activation, classical pathway’ (GO:0,006,958) (Fig. 5a), indicating an altered inflammatory response in the patient’s M2 macrophages. Additional Kyoto Encyclopaedia of Genes and Genomes (KEGG) enrichment analysis [35] (Fig. 5b) showed that, although few KEGG terms were significantly enriched in M0 macrophages (Fig. S5b), terms related to cytokine signalling, particularly ‘NF-kappa B signalling pathway’ (hsa04064), ‘IL-17 signalling pathway’ (hsa04657) and ‘Cytokine-cytokine receptor interaction’ (hsa04060) were enriched in M1 macrophages, the latter of which was also enriched in the patient’s M2 macrophages (Fig. 5b). We then generated a protein–protein interaction network using STRING version 11.0 [36] (Fig. 5c). While the nodes from the M0 DEGs were mostly independent (Fig. S5c), clusters of nodes seen in M1 and M2 macrophages showed prominent involvement of the immune system and cytokine and chemokine signalling, with *CXCL8*, encoding IL-8, acting as a key component of many of the node clusters (Fig. 5c). Together, these analyses highlight a series of inflammatory pathways other than those directly regulated by the NLRP3 inflammasome in this disease state.

## Discussion

The NLRP3 inflammasome is the most widely studied of the inflammasomes, with over 150 currently validated pathogenic mutations [23]. Among these mutations, very few are in the LRR domain, and as a result, our knowledge of *NLRP3*-AIDs caused by such mutations is incomplete. We show here that the *NLRP3*-p.R920Q mutation causes enhanced interactions between NLRP3 and NEK7, leading to increased NLRP3 inflammasome activity and affecting macrophage cytokine signalling.

Mutations in the LRR domain are associated with atypical or milder *NLRP3*-AIDs phenotypes [37], as seen here. Our case report adds to the small number of patients known to carry the *NLRP3*-p.R920Q mutation [5]. In these patients, the primary symptom was sensorineural hearing loss, whereas our patient did not report hearing difficulties. Of the two families already described, one reported no further *NLRP3*-AID symptoms, whereas members of the second family reported autoinflammatory signs and symptoms including oral ulcers but without serologic signs of inflammation. This disparity in symptoms shows that the p.R920Q mutation can be associated with different disease severity in different families, a phenomenon observed with other *NLRP3* mutations [38]. However, arguably, the largest difference between our patient and previously reported families was response to treatment. Anakinra was effective in treating hearing loss and improving the symptoms of autoinflammation in both published families [5]; in contrast, our patient only partially responded to anakinra, which improved her fevers and partly helped with headaches and arthralgia but did not improve mucosal ulceration. Oral ulcers were improved; however, following addition of the PDE4 inhibitor, apremilast, to her treatment regimen, suggesting that signalling pathways other than those directly mediated by the NLRP3 inflammasome were dysregulated. RNA-seq showed that the NF-кB signalling pathway was enriched in the patient’s M1 macrophages, and since apremilast acts via a NF-кB-dependent mechanism to reduce levels of inflammatory cytokines such as TNF [39], this may provide an explanation for the efficacy of this treatment. The IL-17 signalling pathway was also enriched in M1 macrophages, which is notable because NLRP3 inflammasome activation and IL-1β release have been linked to enhanced inflammatory Th17 cell responses in diseases including ankylosing spondylitis [40], inflammatory skin diseases including hidradenitis suppurativa [41, 42], rheumatoid arthritis [43] and obliterative bronchiolitis [44]. A MWS-related *NLRP3* mutation also caused spontaneous skin inflammation in mice due to increased IL-1β production and consequent Th17 cell predominance [45]. As such, enrichment of IL-17 signalling in our patient’s M1 macrophages may contribute to the inflammatory phenotype. It may also partly explain the efficacy of apremilast, as this agent reduces inflammatory cytokine release and increases anti-inflammatory cytokine production in response to IL-17 stimulation in psoriasis [46–48], and IL-17A is a biomarker of apremilast efficacy in psoriasis [49]. *CXCL8*, encoding the neutrophil chemoattractant IL-8, was also upregulated in the patient’s M1 and M2 macrophages. Aberrant regulation of IL-8 has been implicated in numerous inflammatory diseases [50–53], including mucosal tissues, which may partly explain the mucosal ulceration observed here.

The study which first reported pathogenic cases of this mutation also investigated the cochlea of mice and identified tissue-resident macrophage-like cells which secrete IL-1β in response to LPS and ATP stimulation, which may mediate local autoinflammation in the cochlea [5]. In this report, we have expanded on this by elucidating a molecular mechanism of how the p.R920Q mutation leads to an atypical phenotype. Previously reported *NLRP3* mutations have been shown to affect interactions between NLRP3 and its endogenous regulator NEK7 [10, 27]; the p.G755R and p.G755A mutations, which cause CINCA, increase the affinity between NLRP3 and NEK7 [24, 25], while the hypomorphic *NLRP3* missense mutation p.D946G binds with less avidity to NEK7 than to the WT protein [26]. The p.R920Q mutation increases NLRP3 interactions with NEK7, which was predicted by our in silico interrogation of the protein structure to be due to the reduction in positive charge density at the NLRP3/NEK7 interface when the positively charged arginine residue was substituted for an uncharged glutamine.

The function of the LRR domain remains unclear. The recently determined cryo-EM structure of NLRP3 in complex with NEK7 suggests that the LRR and NACHT domains of NLRP3 are important in protein–protein interactions with NEK7 [10], and the second of the two major isoforms, identified in humans lacking exon 5 resulting in a truncated LRR domain, exhibits a loss of activity due to a loss of NLRP3/NEK7 interactions [54]. However, other studies have shown that NLRP3 protein lacking the LRR domain can be activated by the canonical inflammasome pathway [4, 55, 56]. Two recent preprint studies reporting cryo-EM structures of full-length NLRP3 in its native form indicate that interactions between LRR domains are important for the formation of its endogenous ‘ring cage’ structure [57, 58]. In Hochheiser et al., residue R920 is proposed to lie within a concave site in the LRR domain which forms contacts between two opposing LRRs; this LRR-LRR interaction is mediated by an acidic loop extending from an LRR transition segment, suggesting that the p.R920Q mutation may affect electrostatic interactions between this loop and the LRR domain in the inactive form. Overall, our observations add to the LRR story, supporting the idea that missense mutations in the LRR domain can play a crucial role in NLRP3 inflammasome function.

Despite the increased understanding with regards to the structural basis of NLRP3 inflammasome function, efforts in understanding the structure–function relationship of NLRP3 are frequently centred on the central NACHT domain. This is due to a combination of factors, including the majority of the *NLRP3*-AID-related mutations being found in this region [59] and because many NLRP3 inhibitors bind to this domain [60, 61]. The most well-studied pharmacological NLRP3 inflammasome inhibitor, MCC950, has been shown to target NLRP3 in the region of the Walker B motif of the NACHT domain [60]. As such, previous studies utilising a structure-based approach to investigate small molecule inhibitors of the NLRP3 inflammasome, using either a homology model of NLRP3 produced using the NLRC4 crystal structure [62] or the NLRP3/NEK7 cryo-EM structure [10], have targeted the search of druggable binding sites close to the Walker B region [63, 64]. However, the NLRP3 inflammasome inhibitor 3,4-Methylenedioxy-β-nitrostyrene (MNS) binds to the NACHT and LRR domains [65], demonstrating that there is potential for small molecule inhibitors targeting domains other than NACHT, particularly in disease conditions involving LRR domain mutations.

Due to a scarcity of patient blood samples resulting from a combination of her pregnancy and COVID-19 restrictions in hospitals, the present study primarily investigated the effects of the p.R920Q variant in circulating myeloid cells, where the NLRP3 inflammasome is predominantly expressed (Guarda et al. 2011). However, a future avenue of investigation would be to explore the effect of this mutation in cells such as neutrophils, particularly as neutrophil-specific *Nlrp3* mutations have recently been shown to promote the development of *NLRP3*-AIDs in mice [66], and PDE4 inhibitors such as apremilast can inhibit neutrophil extracellular trap formation [67, 68]. This may also offer further insights into the kinetics of the p.R920Q mutation, in addition to the immunoprecipitation studies carried out above.

In summary, we have shown that the rare *NLRP3*-p.R920Q mutation is associated with an atypical presentation. This is likely connected to enhanced NLRP3-NEK7 interactions, which lead to a lower activation threshold for the NLRP3 inflammasome, in addition to involvement of different convergent pathways. Our results highlight the importance of investigating alternative pathways involved in disease states and outline a potential course of treatment for atypical *NLRP3*-AIDs patients.

## Supplementary Information

Below is the link to the electronic supplementary material.

Supplementary file1 (DOCX 1553 KB)

## Data Availability

Data available upon request.
