# Supplementary material for: An Atypical Autoinflammatory Disease Due to an LRR Domain NLRP3 Mutation Enhancing Binding to NEK7
Source: J Clin Immunol. 2021 Oct 21;42(1):158–70. doi: 10.1007/s10875-021-01161-w (PMC8528658; doi:10.1007/s10875-021-01161-w)
Supplement: Supplementary file 1 — Supplementary file1 (DOCX 1553 KB) [file 10875_2021_1161_MOESM1_ESM.docx]

**Supplementary methods**

**Autoinflammatory/autoimmune gene panel for sequencing** Sequencing was performed on a HiSeq 3000 (Illumina) using a 2x150-bp paired-end sequencing protocol. Analysis was restricted to an autoinflammatory/autoimmune gene panel consisting of ACP5, ADAR, AIRE, C1QA, C1QB, C1QC, C3, CARD14, CECR1, CFHR5, CFI, DNASE1, DNASE1L3, FOXP3, ICOS, IFIH1, IL10, IL10RA, IL10RB, IL1RN, IL2RA, IL36RN, IRF8, MEFV, MVK, MYD88, NLRP12, NLRP3, NOD2, PLCG2, PRKCD, PSMB4, PSTPIP1, RAG1, RAG2, RNASEH2A, RNASEH2B, RNASEH2C, SAMHD1, SERPING1, SLC29A3, STAT1, STAT3, TMEM173, TNFAIP3, TNFRSF11A, TNFRSF1A, TREX1 and WDR1.

**Cell culture and stimulation** PBMCs were isolated from whole blood using Lymphoprep density gradient media (StemCell). Whole blood was diluted 50:50 with Ca^2+^ and Mg^2+^ free DPBS supplemented with 2 % heat-inactivated foetal bovine serum (FBS) (Thermo Fisher Scientific), then layered onto Lymphoprep and centrifuged at 1100 x g for 20 min with no brake. The buffy coat was collected and washed twice in DPBS + 2% FBS, and CD14+/CD16- monocytes isolated using an EasySep Human Monocyte Isolation Kit (StemCell). Monocytes were either cultured at a 1 x 10^6^ cells/mL in complete RPMI media (supplemented with 10 % heat inactivated FBS, 50 U/mL penicillin, 50 mg/mL streptomycin and 2 mM L-glutamine) (Merck) or differentiated into macrophages. For macrophage differentiation, monocytes were plated at a density of 0.5 x 10^6^ cells/mL in 1 mL complete RPMI medium supplemented with 20 ng/mL GM-CSF (PeproTech) for 6 days, which was replenished with 500 μL of supplemented media after 3 days. After 6 days, 100 ng/mL IFN-γ was added to M0 macrophages, 50 ng/mL LPS to M1 macrophages, or 20 ng/mL IL-13 and IL-4 (PeproTech) to M2 macrophages, for 24 hr.

**Cytokine quantification by ELISA** Commercially available ELISA kits were sourced from Thermo Fisher Scientific, with product codes IL-1β: CHC1213, IL-18: BMS267-2MST, TNF: CHC1753, IL-6: BMS213-2MST, IL-10: 88-7106.

**ASC speck quantification gating strategy** 2 μL of PE-conjugated ASC antibody (HASC-71 clone, BioLegend) was added to 100 μL of cell culture media and the mixture incubated in FACS collection tubes on a shaker for 1 hr. Size gating was carried out with Megamix-Plus beads (Biocytex) according to the manufacturer’s specifications, as shown in panel A below, and was used to threshold out readings below 0.9 μm, as shown in panel B below; all particles within the gate indicated with an arrow were analysed. Samples were run and analysed on a CytoFLEX-S (Beckman Coulter), where PE-stained particles were determined to be ASC specks in unstimulated and NLRP3 inflammasome stimulated samples (panel C, left and right images, respectively).


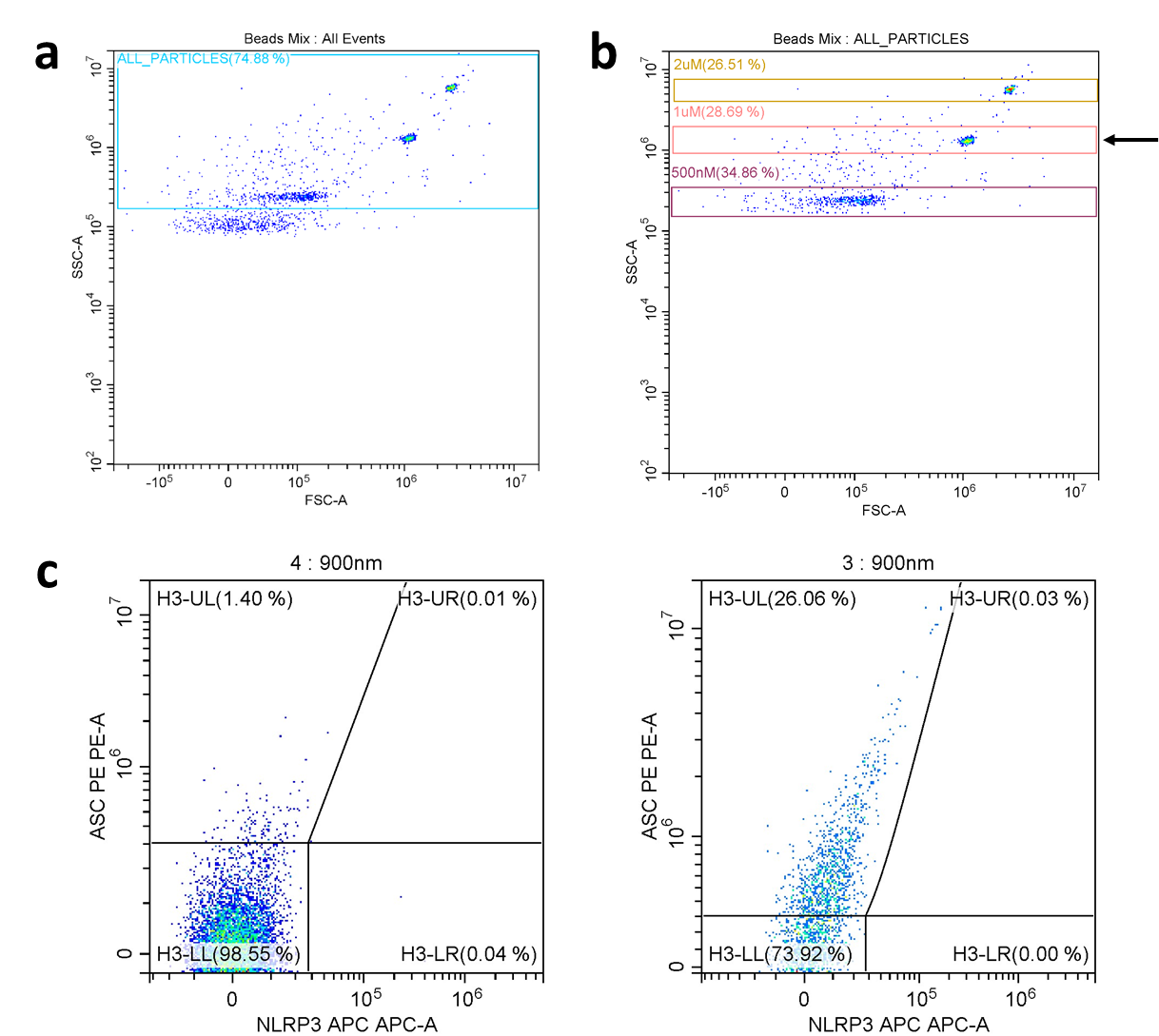


**Gene expression analysis** RNA isolation was carried out using TRIzol Reagent and Phasemaker tubes (Thermo Fisher Scientific) according to the manufacturer’s specifications. RNA quantity and purity were determined using the 260/280 and 260/230 ratios, as determined using a NanoDrop 1000 spectrophotometer (Thermo Fisher Scientific). 100 ng of RNA was converted to cDNA using the SuperScript IV one-step RT-PCR system (Invitrogen) according to the manufacturer’s specifications. Gene expression was analysed using TaqMan probes (Thermo Fisher Scientific) for *IL1B* (Hs01555410_m1), *IL18* (Hs01038788_m1), *TNF* (Hs00174128_m1), *IL6* (Hs00174131_m1), *IL10* (Hs00961622_m1), *NLRP3* (Hs00918082_m1), *CASP1* (Hs00354836_m1), *PYCARD* (Hs01547324_gH), *ACTB* (Hs03023943_g1), *HPRT1* (Hs02800695_m1), in a reaction containing 100 ng of cDNA, 1 μL TaqMan gene expression assay, 10 μL TaqMan gene expression Master Mix (Thermo Fisher Scientific) and nuclease-free water to a final volume of 20 μL. Cycle parameters were 50⁰C for 2 min then 95⁰C for 10 min, followed by 40 cycles of 95⁰C for 15 sec and 60⁰C for 1 min; samples were processed in duplicate on a QuantStudio 7 Flex Real-Time PCR System (Applied Biosystems). Data were expressed as relative expression compared to the housekeeping genes, *ACTB* and *HPRT1.*

**RNAseq** RNA Library preparation and RNA sequencing (RNA-seq) was carried out on a high-throughput Illumina platform and paired-end reads generated (Novogene (UK) Company Limited). An average of 22.3 million raw reads were generated per sample (Q30 average of 94.1%). Raw reads were trimmed using fastp[1] to remove reads containing adapter and poly-N sequences and low quality reads (QV ≤ 30). Paired-end clean reads were mapped to the reference genome, Homo sapiens GRCh38 (hg38), using the Spliced Transcripts Alignment to a Reference (STAR) software[2]. Differential expression analysis was performed using the DESeq2 R package[3]. M0, M1 and M2 macrophages showed an average of 92.4 %, 90.3 % and 92.4 % uniquely mapped reads, respectively. Gene ontology (GO) and Kyoto Encyclopedia of Genes and Genomes (KEGG) enrichment analysis of differentially expressed genes was implemented by clusterProfiler[4]. GO/KEGG terms with corrected P value of less than 0.05 were considered significantly enriched.

**Immunoprecipitation studies** 3 x 10^6^ HEK293T cells were plated in a 10 mL petri dish in 10 mL complete DMEM medium 24 hours before transfection. Cells were transfected with 10 μg of plasmids for NLRP3-GFP, NEK7-His using Opti-MEM and Lipofectamine 2000 (Thermo Fisher Scientific) according to the manufacturer’s specifications, then incubated at 37⁰C for 36 hours. Cells were washed twice with ice cold PBS and harvested by scraping into 1 mL of ice-cold PBS and centrifuging at 500 x g for 2 min. The supernatant was aspirated and cells resuspended in 257 μL of resuspension buffer (10 mM Hepes-KOH pH 7.9, 10 mM HCl, 1.5 mM MgCl_2_ and 0.1% NP-40) supplemented with protease cOmplete Mini Protease Inhibitor Cocktail Tablets (Roche). 18 μL of 5 M NaCl added to the lysate, followed by 25 μL 40 % glycerol, and the lysate rotated at 4⁰C for 20 min. The lysate was centrifuged at 20,000 x g for 15 min, and the resulting supernatant added to 300 μL of 10 mM Hepes pH 7.9 supplemented with protease inhibitors, then pre-cleared by adding 15 μL of Pierce protein A agarose (Thermo Fisher Scientific) and rotating at 4⁰C for 20 min. Pre-cleared lysate was added to 1 μg of anti-NLRP3 antibody (Cryo-2, AdipoGen) and the mixture rotated overnight at 4⁰C, following which 50 μL of equilibrated protein A agarose beads were added and the lysate mixed at 4⁰C for 1 hr. Agarose beads were centrifuged at 500 x g 4⁰C for 2 min, then washed 3 times with suspension buffer (10 mM Hepes pH 7.9, 5 mM KCl, 0.75 mM MgCl_2_, 150 mM NaCl, 0.05 % NP-40) supplemented with protease inhibitors. The pellet was resuspended in 40 μL resuspension buffer, mixed with 5 x protein loading buffer, boiled for 5 minutes, and analysed by SDS-PAGE and Western blot.

**Western blot** Protein levels were determined by Western blot. Samples were loaded alongside a PageRuler™ protein ladder (Thermo Fisher Scientific) and electrophoresed on 4-15 % Mini-PROTEAN® gels (Bio-Rad). Proteins were subsequently transferred onto PVDF membranes (Bio-Rad) by wet transfer in transfer buffer (25 mM Tris, 192 mM glycine, pH 8.3, 20% methanol) at 100 V for 70 min, then blocked with phosphate buffered saline with 0.1 % Tween 20 (PBST) containing 5 % w/v non-fat milk at RT for 1 hr. Membranes were cut to allow simultaneous staining with different antibodies, before hybridisation with the relevant antibody: rabbit anti-His (PA1-983B, Thermo Fisher Scientific) diluted 1:1000, or rabbit anti-GFP (orb195989, Biorbyt) diluted 1:1000 overnight at 4 ⁰C. Membranes were washed 3 times in PBST and incubated with goat anti-rabbit IgG (H+L) poly-HRP secondary antibody (ThermoFisher Scientific) diluted 1:5000 in PBST with 5% milk for 1 hr at RT. Membranes were washed 5 times with PBST and proteins detected by the addition of 5 mL of ECL detection system (Merck) for 1 min, followed by imaging using a ChemiDoc™ Touch system (Bio-Rad).

**Plasmids** The human NEK7 expression plasmid in a pCMV3 vector with a C-terminal His tag was sourced from Sino Biological (catalogue number HG11534-CH), coding region sequence: ATGGATGAGCAATCACAAGGAATGCAAGGGCCACCTGTTCCTCAGTTCCAACCACAGAAGGCCTTACGACCGGATATGGGCTATAATACATTAGCCAACTTTCGAATAGAAAAGAAAATTGGTCGCGGACAATTTAGTGAAGTTTATAGAGCAGCCTGTCTCTTGGATGGAGTACCAGTAGCTTTAAAAAAAGTGCAGATATTTGATTTAATGGATGCCAAAGCACGTGCTGATTGCATCAAAGAAATAGATCTTCTTAAGCAACTCAACCATCCAAATGTAATAAAATATTATGCATCATTCATTGAAGATAATGAACTAAACATAGTTTTGGAACTAGCAGATGCTGGCGACCTATCCAGAATGATCAAGCATTTTAAGAAGCAAAAGAGGCTAATTCCTGAAAGAACTGTTTGGAAGTATTTTGTTCAGCTTTGCAGTGCATTGGAACACATGCATTCTCGAAGAGTCATGCATAGAGATATAAAACCAGCTAATGTGTTCATTACAGCCACTGGGGTGGTAAAACTTGGAGATCTTGGGCTTGGCCGGTTTTTCAGCTCAAAAACCACAGCTGCACATTCTTTAGTTGGTACGCCTTATTACATGTCTCCAGAGAGAATACATGAAAATGGATACAACTTCAAATCTGACATCTGGTCTCTTGGCTGTCTACTATATGAGATGGCTGCATTACAAAGTCCTTTCTATGGTGACAAAATGAATTTATACTCACTGTGTAAGAAGATAGAACAGTGTGACTACCCACCTCTTCCTTCAGATCACTATTCAGAAGAACTCCGACAGTTAGTTAATATGTGCATCAACCCAGATCCAGAGAAGCGACCAGACGTCACCTATGTTTATGACGTAGCAAAGAGGATGCATGCATGCACTGCAAGCAGCGGGGGTGGAGGCTCTCACCATCACCACCATCATCACC

The human NLRP3 expression plasmid in a pEZ-M03 vector with a C-terminal GFP tag was sourced from Genecopoeia (catalogue number EX-E1447-M03), coding region sequence: TAAGGAGTTTAAACACCATGAAGATGGCAAGCACCCGCTGCAAGCTGGCCAGGTACCTAGAGGACCTGGAGGATGTGGACTTGAAGAAATTTAAGATGCACTTAGAGGACTATCCTCCCCAGAAGGGCTGCATCCCCCTCCCGAGGGGTCAGACAGAGAAGGCAGACCATGTGGATCTAGCCACGCTAATGATCGACTTCAATGGGGAGGAGAAGGCGTGGGCCATGGCCGTGTGGATCTTCGCTGCGATCAACAGGAGAGACCTTTATGAGAAAGCAAAAAGAGATGAGCCGAAGTGGGGTTCAGATAATGCACGTGTTTCGAATCCCACTGTGATATGCCAGGAAGACAGCATTGAAGAGGAGTGGATGGGTTTACTGGAGTACCTTTCGAGAATCTCTATTTGTAAAATGAAGAAAGATTACCGTAAGAAGTACAGAAAGTACGTGAGAAGCAGATTCCAGTGCATTGAAGACAGGAATGCCCGTCTGGGTGAGAGTGTGAGCCTCAACAAACGCTACACACGACTGCGTCTCATCAAGGAGCACCGGAGCCAGCAGGAGAGGGAGCAGGAGCTTCTGGCCATCGGCAAGACCAAGACGTGTGAGAGCCCCGTGAGTCCCATTAAGATGGAGTTGCTGTTTGACCCCGATGATGAGCATTCTGAGCCTGTGCACACTGTGGTGTTCCAGGGGGCGGCAGGGATTGGGAAAACAATCCTGGCCAGGAAGATGATGTTGGACTGGGCGTCGGGGACACTCTACCAAGACAGGTTTGACTATCTGTTCTATATCCACTGTCGGGAGGTGAGCCTTGTGACACAGAGGAGCCTGGGGGACCTGATCATGAGCTGCTGCCCCGACCCAAACCCACCCATCCACAAGATCGTGAGAAAACCCTCCAGAATCCTCTTCCTCATGGACGGCTTCGATGAGCTGCAAGGTGCCTTTGACGAGCACATAGGACCGCTCTGCACTGACTGGCAGAAGGCCGAGCGGGGAGACATTCTCCTGAGCAGCCTCATCAGAAAGAAGCTGCTTCCCGAGGCCTCTCTGCTCATCACCACGAGACCTGTGGCCCTGGAGAAACTGCAGCACTTGCTGGACCATCCTCGGCATGTGGAGATCCTGGGTTTCTCCGAGGCCAAAAGGAAAGAGTACTTCTTCAAGTACTTCTCTGATGAGGCCCAAGCCAGGGCAGCCTTCAGTCTGATTCAGGAGAACGAGGTCCTCTTCACCATGTGCTTCATCCCCCTGGTCTGCTGGATCGTGTGCACTGGACTGAAACAGCAGATGGAGAGTGGCAAGAGCCTTGCCCAGACATCCAAGACCACCACCGCGGTGTACGTCTTCTTCCTTTCCAGTTTGCTGCAGCCCCGGGGAGGGAGCCAGGAGCACGGCCTCTGCGCCCACCTCTGGGGGCTCTGCTCTTTGGCTGCAGATGGAATCTGGAACCAGAAAATCCTGTTTGAGGAGTCCGACCTCAGGAATCATGGACTGCAGAAGGCGGATGTGTCTGCTTTCCTGAGGATGAACCTGTTCCAAAAGGAAGTGGACTGCGAGAAGTTCTACAGCTTCATCCACATGACTTTCCAGGAGTTCTTTGCCGCCATGTACTACCTGCTGGAAGAGGAAAAGGAAGGAAGGACGAACGTTCCAGGGAGTCGTTTGAAGCTTCCCAGCCGAGACGTGACAGTCCTTCTGGAAAACTATGGCAAATTCGAAAAGGGGTATTTGATTTTTGTTGTACGTTTCCTCTTTGGCCTGGTAAACCAGGAGAGGACCTCCTACTTGGAGAAGAAATTAAGTTGCAAGATCTCTCAGCAAATCAGGCTGGAGCTGCTGAAATGGATTGAAGTGAAAGCCAAAGCTAAAAAGCTGCAGATCCAGCCCAGCCAGCTGGAATTGTTCTACTGTTTGTACGAGATGCAGGAGGAGGACTTCGTGCAAAGGGCCATGGACTATTTCCCCAAGATTGAGATCAATCTCTCCACCAGAATGGACCACATGGTTTCTTCCTTTTGCATTGAGAACTGTCATCGGGTGGAGTCACTGTCCCTGGGGTTTCTCCATAACATGCCCAAGGAGGAAGAGGAGGAGGAAAAGGAAGGCCGACACCTTGATATGGTGCAGTGTGTCCTCCCAAGCTCCTCTCATGCTGCCTGTTCTCATGGATTGGTGAACAGCCACCTCACTTCCAGTTTTTGCCGGGGCCTCTTTTCAGTTCTGAGCACCAGCCAGAGTCTAACTGAATTGGACCTCAGTGACAATTCTCTGGGGGACCCAGGGATGAGAGTGTTGTGTGAAACGCTCCAGCATCCTGGCTGTAACATTCGGAGATTGTGGTTGGGGCGCTGTGGCCTCTCGCATGAGTGCTGCTTCGACATCTCCTTGGTCCTCAGCAGCAACCAGAAGCTGGTGGAGCTGGACCTGAGTGACAACGCCCTCGGTGACTTCGGAATCAGACTTCTGTGTGTGGGACTGAAGCACCTGTTGTGCAATCTGAAGAAGCTCTGGTTGGTCAGCTGCTGCCTCACATCAGCATGTTGTCAGGATCTTGCATCAGTATTGAGCACCAGCCATTCCCTGACCAGACTCTATGTGGGGGAGAATGCCTTGGGAGACTCAGGAGTCGCAATTTTATGTGAAAAAGCCAAGAATCCACAGTGTAACCTGCAGAAACTGGGGTTGGTGAATTCTGGCCTTACGTCAGTCTGTTGTTCAGCTTTGTCCTCGGTACTCAGCACTAATCAGAATCTCACGCACCTTTACCTGCGAGGCAACACTCTCGGAGACAAGGGGATCAAACTACTCTGTGAGGGACTCTTGCACCCCGACTGCAAGCTTCAGGTGTTGGAATTAGACAACTGCAACCTCACGTCACACTGCTGCTGGGATCTTTCCACACTTCTGACCTCCAGCCAGAGCCTGCGAAAGCTGAGCCTGGGCAACAATGACCTGGGCGACCTGGGGGTCATGATGTTCTGTGAAGTGCTGAAACAGCAGAGCTGCCTCCTGCAGAACCTGGGGTTGTCTGAAATGTATTTCAATTATGAGACAAAAAGTGCGTTAGAAACACTTCAAGAAGAAAAGCCTGAGCTGACCGTCGTCTTTGAGCCTTCTTGGTACCTCGAGTGCGGCCGC

**Supplementary figures**


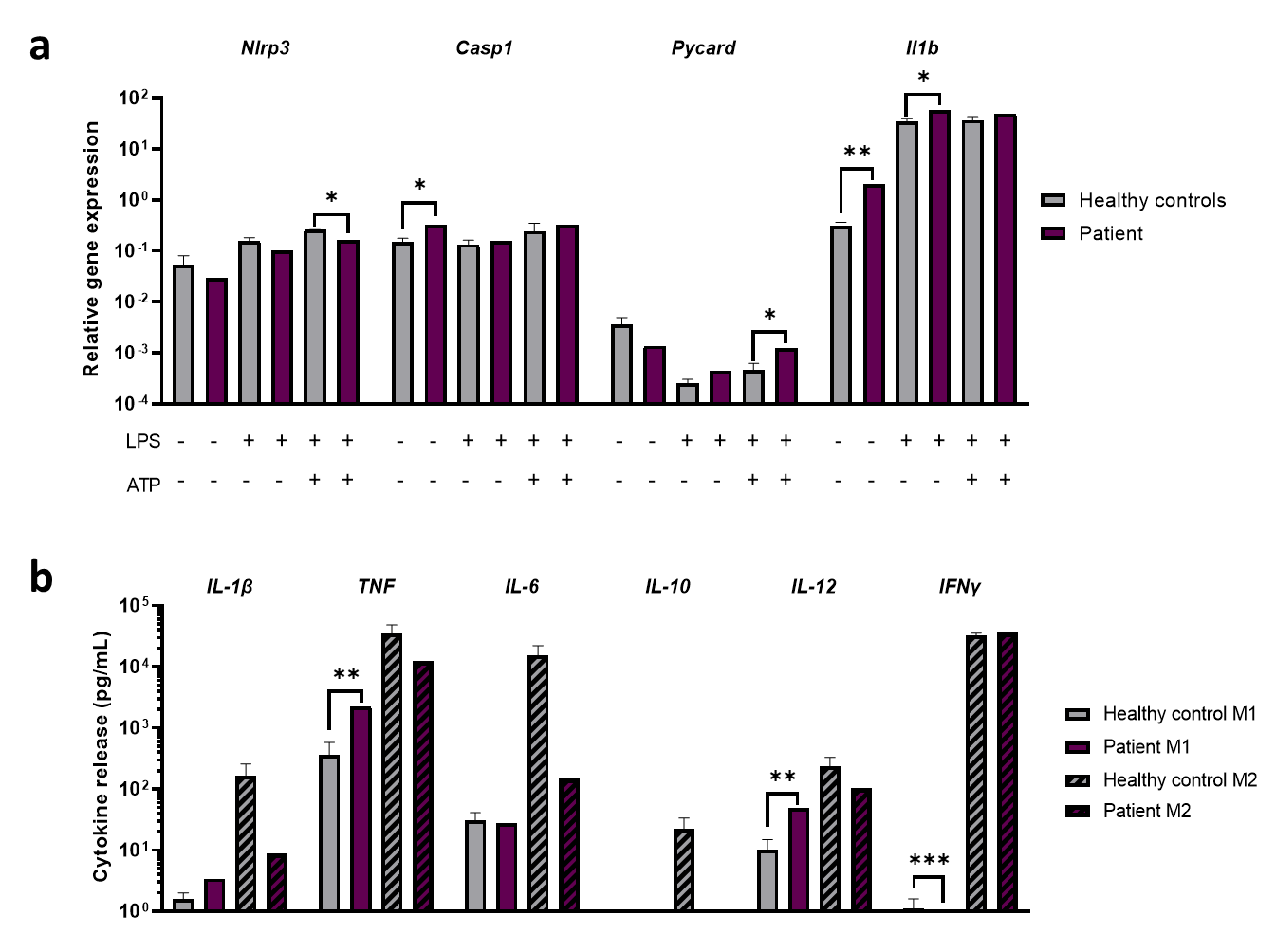


**Figure S1. Monocyte and macrophage inflammatory profile suggest involvement of additional inflammatory pathways.** (A) mRNA relative expression of NLRP3 inflammasome genes in the patient’s primary monocytes when unstimulated, stimulated with LPS alone, or with LPS and ATP for NLRP3 inflammasome activation, compared to 3 HCs. (B) Cytokine profile of the patient’s unstimulated M1 and M2 macrophages, compared to 2 HCs. *p < 0.05, **p < 0.01, ***p < 0.001.


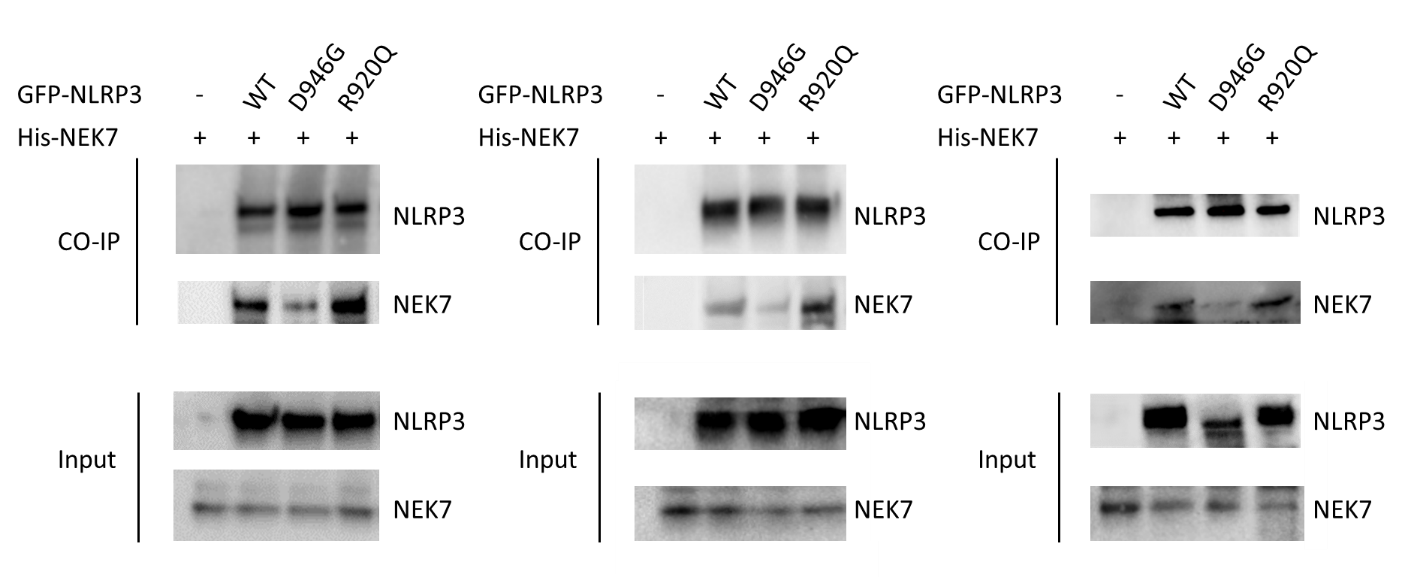


**Figure S2. Repeat of the co-immunoprecipitation experiments using HEK293T cells transfected with WT NLRP3, or NLRP3 with the p.R920Q or p.D946G mutations.**


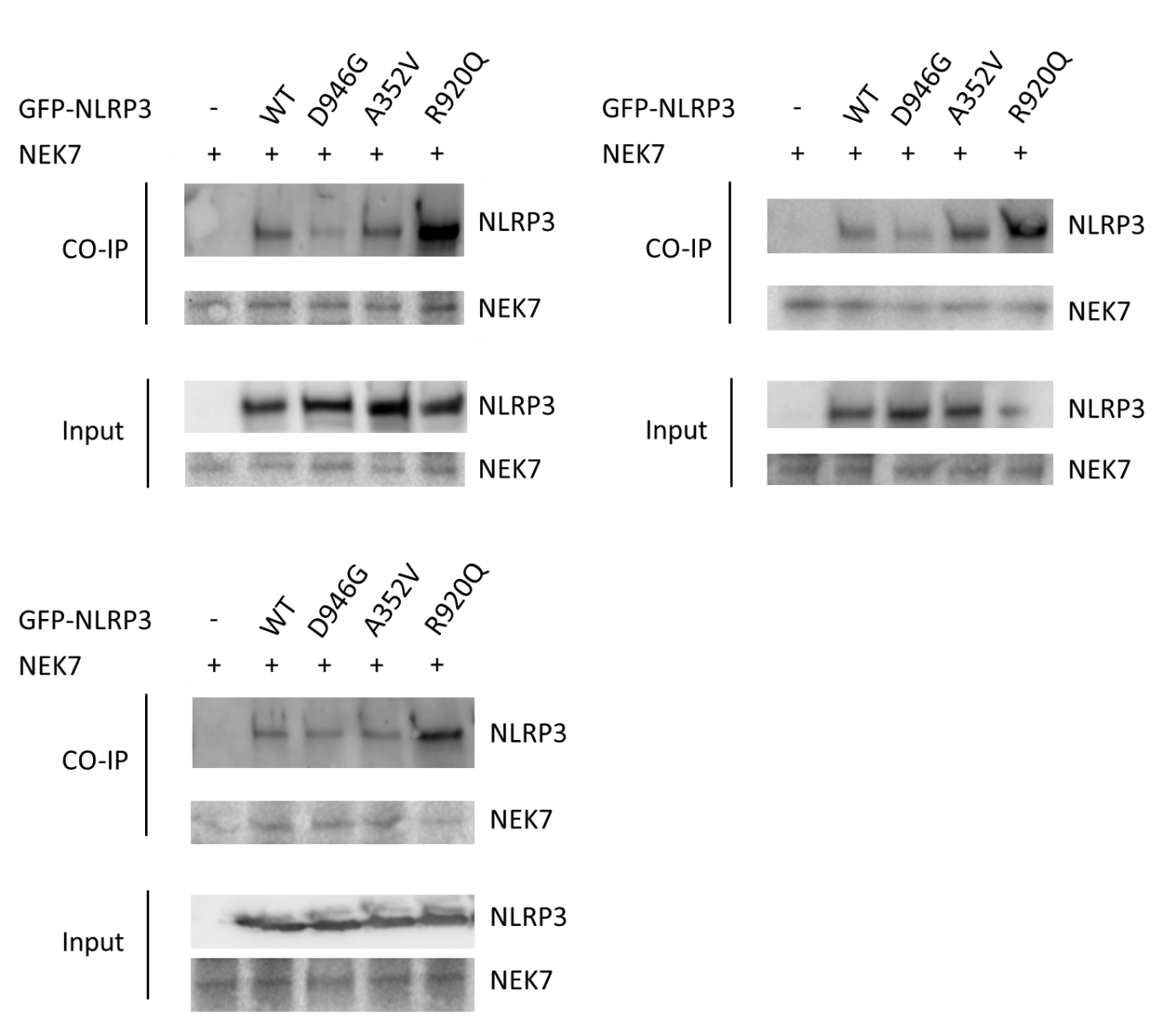


**Figure S3. Repeat of the co-immunoprecipitation experiments using HEK293T cells** **expressing endogenous NEK7 and transfected with WT NLRP3, or NLRP3 with the p.D946G, p.A352V or p.R920Q mutations.**

**
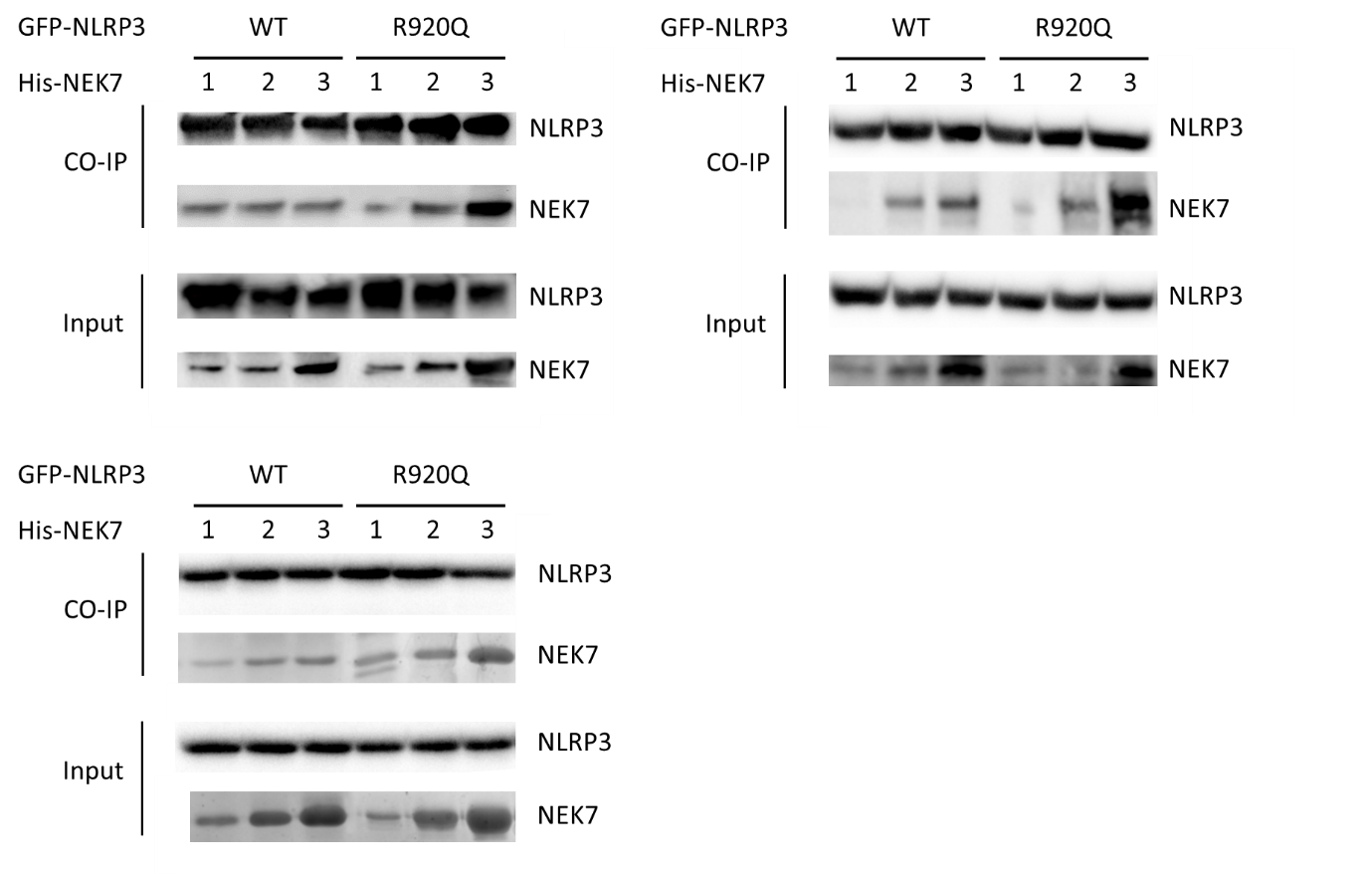
**

**Figure S4. Repeat of the co-immunoprecipitation experiments using HEK293T cells** **expressing endogenous NEK7 and transfected with WT or p.R920Q-NLRP3 with increasing concentrations of His-tagged NEK7; 25 %, 50 %, or 100 % as indicated.**


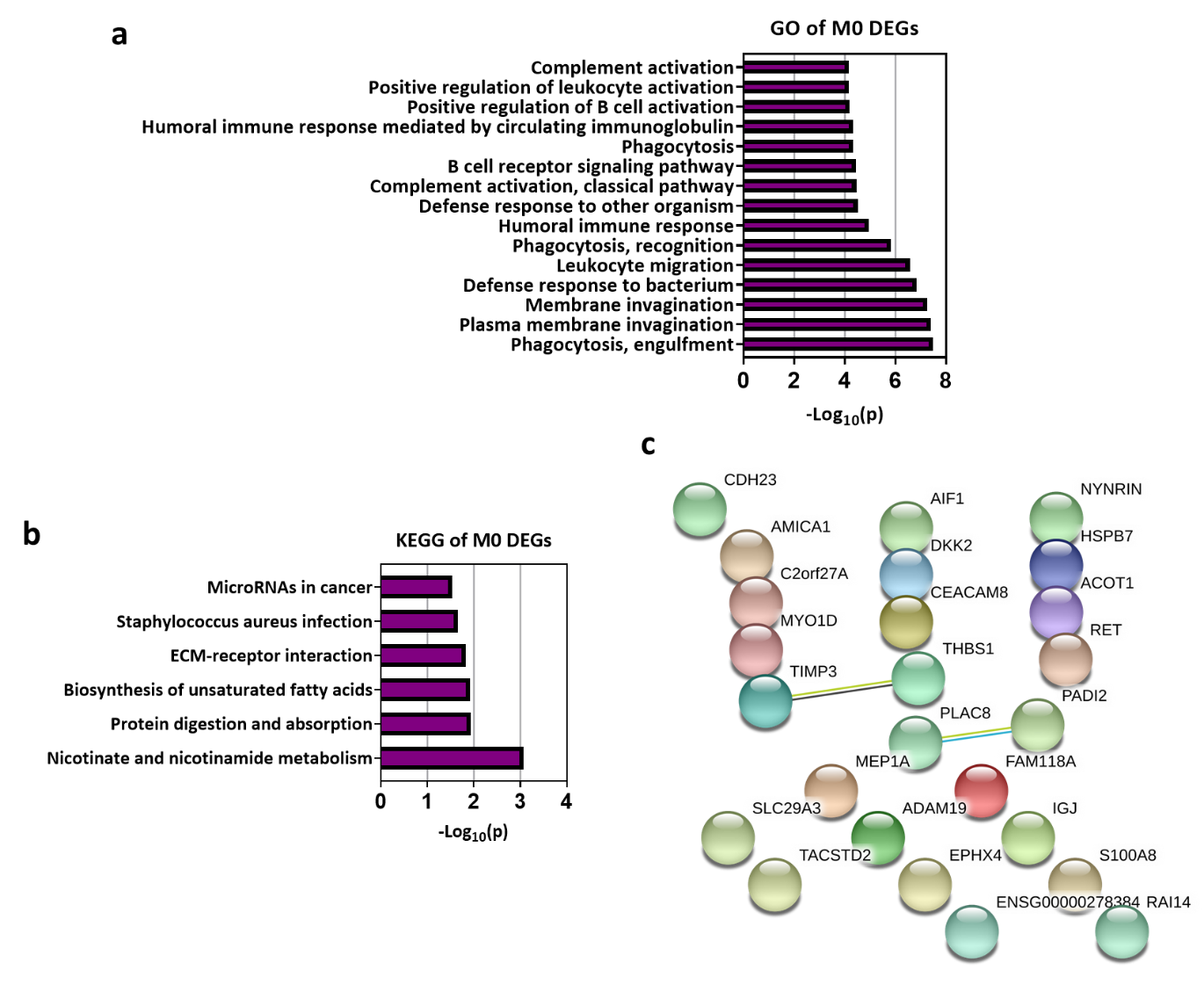


**Figure S5. Gene set enrichment analyses show less distinct pathway enrichment in M0 macrophages compared to M1 and M2 macrophages.** (A) GO enrichment analysis of DEGs between the patient’s and HC M0 macrophages showing the 15 most significant GO terms (p < 0.001). (B) KEGG analysis showing biological function and pathway enrichment of DEGs between the patient and HCs for M0 macrophages (p < 0.05). (C) Network of predicted protein-protein interactions derived from the list of DEGs between the patient’s and HC M0 macrophages, as determined by STRING analysis. Lines between nodes depict known interactions from curated databases (light blue), experimentally determined interactions (pink), co-expressed proteins (black) and homologous proteins (violet).

**References**

1. Chen S, Zhou Y, Chen Y, Gu J. fastp: an ultra-fast all-in-one FASTQ preprocessor. Bioinformatics. 2018;34(17):i884-i90.

2. Dobin A, Davis CA, Schlesinger F, Drenkow J, Zaleski C, Jha S, et al. STAR: ultrafast universal RNA-seq aligner. Bioinformatics. 2013;29(1):15-21.

3. Love MI, Huber W, Anders S. Moderated estimation of fold change and dispersion for RNA-seq data with DESeq2. Genome biology. 2014;15(12):1-21.

4. Yu G, Wang L-G, Han Y, He Q-Y. clusterProfiler: an R package for comparing biological themes among gene clusters. Omics: a journal of integrative biology. 2012;16(5):284-7.
